# Selective inhibition of HDAC8 decreases neuroblastoma growth *in vitro* and *in vivo* and enhances retinoic acid-mediated differentiation

**DOI:** 10.1038/cddis.2015.24

**Published:** 2015-02-19

**Authors:** I Rettig, E Koeneke, F Trippel, W C Mueller, J Burhenne, A Kopp-Schneider, J Fabian, A Schober, U Fernekorn, A von Deimling, H E Deubzer, T Milde, O Witt, I Oehme

**Affiliations:** 1 Clinical Cooperation Unit Pediatric Oncology, German Cancer Research Center (DKFZ), Heidelberg, Germany; 2 Department of Pediatric Surgery, Dr. von Hauner Children's Hospital, Ludwig-Maximilians-University Munich, Munich, Germany; 3 Department of Neuropathology, Institute for Pathology, University of Leipzig, Leipzig, Germany; 4 Department of Clinical Pharmacology and Pharmacoepidemiology, University Hospital Heidelberg, Heidelberg, Germany; 5 Department of Biostatistics, German Cancer Research Center (DKFZ), Heidelberg, Germany; 6 Ilmenau University of Technology, Institute for Micro- and Nanotechnologies, Ilmenau, Germany; 7 Department of Neuropathology, Institute of Pathology, Ruprecht-Karls-University Heidelberg, and Clinical Cooperation Unit Neuropathology, German Cancer Research Center (DKFZ), Heidelberg, Germany; 8 Department of Pediatric Oncology, Hematology and Immunology, University Hospital Heidelberg, Heidelberg, Germany

## Abstract

For differentiation-defective malignancies, compounds that modulate transcription, such as retinoic acid and histone deacetylase (HDAC) inhibitors, are of particular interest. HDAC inhibitors are currently under investigation for the treatment of a broad spectrum of cancer diseases. However, one clinical drawback is class-specific toxicity of unselective inhibitors, limiting their full anticancer potential. Selective targeting of individual HDAC isozymes in defined tumor entities may therefore be an attractive alternative treatment approach. We have previously identified HDAC family member 8 (HDAC8) as a novel target in childhood neuroblastoma. Using small-molecule inhibitors, we now demonstrate that selective inhibition of HDAC8 exhibits antineuroblastoma activity without toxicity in two xenograft mouse models of MYCN oncogene-amplified neuroblastoma. In contrast, the unselective HDAC inhibitor vorinostat was more toxic in the same models. HDAC8-selective inhibition induced cell cycle arrest and differentiation *in vitro* and *in vivo*. Upon combination with retinoic acid, differentiation was significantly enhanced, as demonstrated by elongated neurofilament-positive neurites and upregulation of NTRK1. Additionally, MYCN oncogene expression was downregulated *in vitro* and tumor cell growth was markedly reduced *in vivo*. Mechanistic studies suggest that cAMP-response element-binding protein (CREB) links HDAC8- and retinoic acid-mediated gene transcription. In conclusion, HDAC-selective targeting can be effective in tumors exhibiting HDAC isozyme-dependent tumor growth *in vivo* and can be combined with differentiation-inducing agents.

The development of neuroblastoma, the most common extracranial solid tumor of childhood, is hypothesized to be related to maturation defects of neural crest-derived precursor cells of the peripheral sympathetic nervous system. The long-term overall survival probability of high-risk neuroblastoma patients is <50%. In addition, chemotherapy-treated patients struggle with therapy-related immediate and long-term toxicities (reviewed in Brodeur^1^). Thus, more neuroblastoma-optimized therapy approaches aiming at oncogenic molecular targets, for example, affecting neuroblastoma cell maturation and proliferation, are believed to improve therapeutic efficacy, reduce toxicity and avoid long-term side effects.^2^ Small molecules, which influence gene transcription, for example, histone deacetylase (HDAC) inhibitors, are known to induce maturation of differentiation-defective tumor cells, such as neuroblastoma cells (reviewed in Witt *et al.*^3^).

The human family of HDACs is grouped into four classes based on their homology to yeast HDACs. Three of the four classes (class I, II and IV) have a zinc-dependent catalytic mechanism and are the so-called classical HDACs. HDAC family member 8 (HDAC8) together with HDAC1, 2 and 3 compose class I.^4^ HDACs remove acetyl groups from lysine residues of numerous nuclear and cytosolic proteins, affecting gene transcription as well as many cellular pathways (Choudhary *et al.*^5^ and Glozak *et al.*^6^ and reviewed in Marks *et al.*^7^). Small-molecule inhibitors of HDAC enzymatic activity bind to the highly conserved catalytic domain and hence unselectively inhibit the activity of all zinc-dependent HDACs. Vorinostat (SAHA: suberoylanilide hydroxamic acid) was the first HDAC inhibitor to be approved for clinical use by the FDA for the treatment of refractory cutaneous T-cell lymphoma.^8^ In several clinical trials, vorinostat was active against leukemia and lymphoma, but rather modestly effective against solid tumors.^9^ As HDACs are involved in numerous essential cellular processes, the clinical use of broad-spectrum HDAC inhibitors is associated with dose-limiting side effects such as thrombocytopenia, fatigue, nausea, diarrhea and anorexia (reviewed in Lane and Chabner^9^and Witt *et al.*^10^). Because similar toxicity profiles have been independently observed in many clinical trials involving HDAC inhibitors, it is believed that these are drug class-specific side effects, which limit their full anticancer potential. Thus, there is an ongoing debate in the field of HDAC inhibitor development as to whether selective inhibitors targeting only a single HDAC family member that drives tumor growth and survival would result in a larger therapeutic window and greater efficacy compared with broad-spectrum inhibitors currently in clinical use.^11^ So far, *in vivo* proof of concept demonstrating that superior antitumoral activity using an HDAC-isozyme-selective inhibitor can be achieved is lacking. In this regard, HDAC family member 8 appears to be an attractive target, as crystal structure analysis revealed a unique second metal binding site in close proximity to the main catalytic domain,^12^ which distinguishes this HDAC isozyme from the other classical deacetylases. This finding led to the design of linkerless hydroxamic acid-based inhibitors^13, 14^ that fit in this secondary pocket and display a much higher selectivity for HDAC8 over the other classical HDACs. We have previously shown that the expression of HDAC8 correlates with advanced tumor stage and poor outcome in neuroblastoma.^15^ Here, we verify small-molecule inhibition of HDAC8 as a novel therapeutic strategy alone and as a potent enhancer of retinoic acid-mediated differentiation and propose the cAMP-response element-binding protein (CREB) as a link between HDAC8 and retinoic acid-mediated gene transcription.

## Results

### HDAC8 as a neuroblastoma drug target *in vivo*

**Figure 1:**
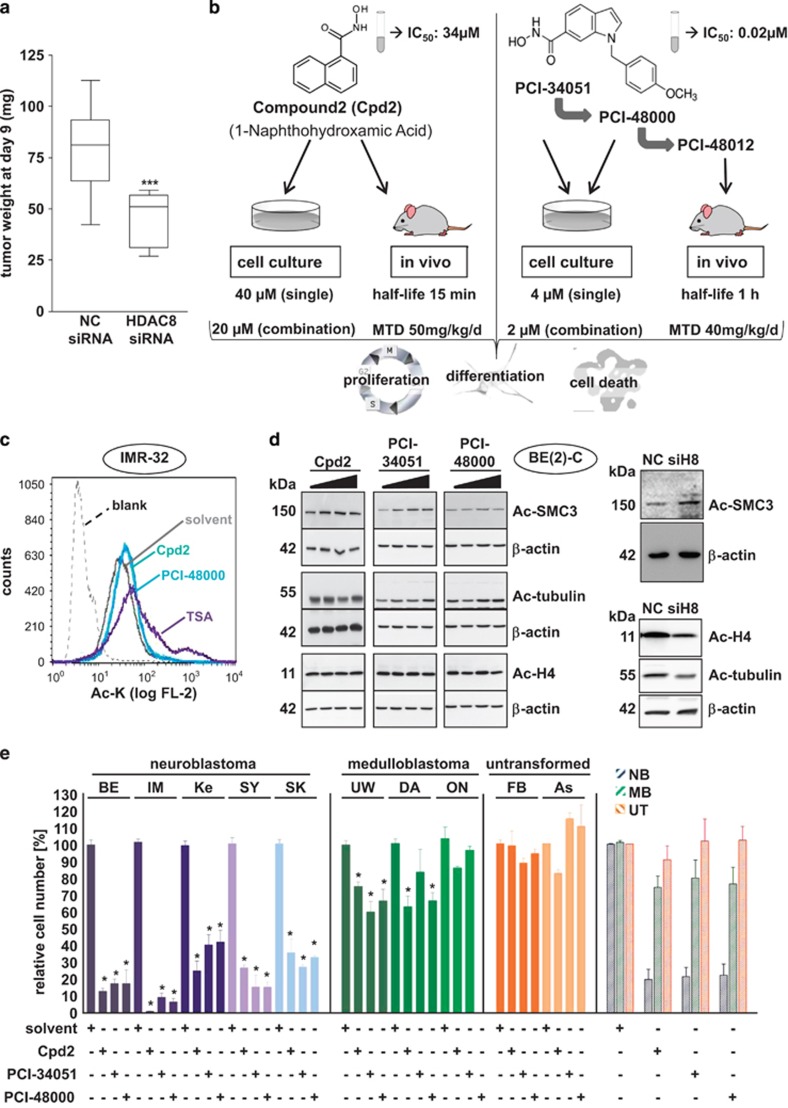
Characterization of HDAC8 inhibitors *in vitro*. (**a**) *In vivo* validation of HDAC8 as a drug target. Tumor weight of explanted BE(2)-C neuroblastoma xenografts after siRNA-mediated transient knockdown of HDAC8 or negative control (NC) in NMRI Foxn1 nude mice (cohorts of 20 animals each). Cells were transplanted 48 h after knockdown. Data are represented as mean tumor weight ± S.E.M. Mann Whitney was used for statistics (***P<0.001). (**b**) Summary of the HDAC8 inhibitors used in the study. The chemical structures of linkerless hydroxamic acid-based inhibitors selective for HDAC8, Cpd2 and PCI-34051 are shown. PCI-48000 and PCI-48012 are more stable variants of PCI-34051. (**c**) Flow cytometric analysis of intracellular whole lysine acetylation levels of IMR-32 cells. Cells were treated with HDAC8 inhibitors Cpd2 (40 *μ*M) and PCI-48000 (4 *μ*M) for 24 h. TSA (trichostatin A, 150 nM) was used as positive control. Solvent: DMSO control. Blank: secondary antibody only. (**d**) Left panel: western blot analysis of histone 4, *α*-tubulin and SMC3 acetylation levels in BE(2)-C cells treated with increasing concentrations of Cpd2 (0, 40, 100 and 200 *μ*M), PCI-34051 (0, 4, 30 and 100 *μ*M) and PCI-48000 (0, 4, 20 and 100 *μ*M). Actin served as a loading control. Right panel: Western blot analysis of histone 4 acetylation, *α*-tubulin acetylation and SMC3 acetylation in HDAC8-depleted (siH8) *versus* negative control siRNA (NC)-transfected BE(2)-C cells. (**e**) Determination of total cell numbers 6 days after treatment of neuroblastoma cell lines (MYCN amplified: BE=BE(2)-C, IM=IMR-32 and Ke=Kelly; non-amplified: SY=SH-SY5Y and SK=SK-N-AS), medulloblastoma cell lines (UW=UW-288-2, DA=DAOY and ON=ONS76) and proliferative infant fibroblasts (FB), as well as astrocytes (As) with HDAC8 inhibitors Cpd2 (40 *μ*M), PCI-34051 (4 *μ*M) and PCI-48000 (4 *μ*M). Cell count was normalized to solvent-treated cells. *T*-test was used for statistics. *P<0.0001, bars represent mean values and error bars represent S.E.M.

We have recently demonstrated that knockdown and inhibition of HDAC8 in neuroblastoma cell cultures induced cell cycle arrest and differentiation.^15^ The aim of the current study was to evaluate HDAC8 as a potential drug target *in vivo*. Before this, we investigated HDAC8 expression in human tumor samples. We reanalyzed publically available expression data from the AMC (Academic Medical Center) cohort (GEO accession no. GSE16476) of neuroblastoma patients using the web-based R2 microarray database (http://r2.amc.nl).^16^ The expression of HDAC8 significantly differed between the tumor stages. HDAC8 expression levels significantly correlated with INSS (International Neuroblastoma Staging System) stage 4 and poor overall survival (Supplementary Figures 1A and B). These results confirm our previously published observation with human tumor samples from the German neuroblastoma trial.^15^ We additionally tested the HDAC8 protein expression in a diversity of neuroblastoma cell lines. All neuroblastoma cell lines used were HDAC8 positive, independent of MYCN oncogene amplification status. In contrast, infant fibroblasts displayed much lower HDAC8 protein levels (Supplementary Figure S1C). To confirm HDAC8 as a potential therapeutic target *in vivo*, we examined the influence of HDAC8 knockdown on tumor growth in a neuroblastoma xenograft mouse model. For this purpose, we used BE(2)-C, a well-established cell line representing a relatively chemotherapy-resistant, highly aggressive, MYCN-amplified and p53 mutant neuroblastoma cell model. Cells transiently transfected with either negative control or HDAC8 targeting siRNA were xenografted subcutaneously into athymic mice. The specificity of the used siRNA was previously defined.^15^ HDAC8 depletion delayed tumor growth with a significant difference in tumor weight at day 9 after tumor transplantation (Figure 1a). *HDAC8* was clearly depleted at the beginning. However, owing to the transient nature of the transfection, the expression of *HDAC8* increased again over time (Supplementary Figure S1D). This result supported our hypothesis to use HDAC8 as a neuroblastoma drug target *in vivo*.

### HDAC8-selective inhibitors display antineuroblastoma activity *in vitro*

To inhibit specifically HDAC8 activity in neuroblastoma, we decided to use and compare two structurally divergent HDAC8 inhibitors, which are both reported to be selective for HDAC8: Compound 2 (Cpd2; 1-naphthohydroxamic acid^13^) and PCI-34051.^14^ For PCI-34051, two more stable variants exist: PCI-48000 and PCI-48012. A comparison of the compounds and concentrations applied is shown in Figure 1b. We confirmed HDAC8 selectivity of Cpd2 and PCI-34051 using *in vitro* cell-free biochemical assays of all classical HDACs 1–11 (Supplementary Table 1). This determined HDAC6 and HDAC1 as potential off-target HDACs at concentrations above 75 *μ*M for Cpd2 and above 30 *μ*M for PCI-34051. Thus, acetylation levels of HDAC6 or HDAC1 substrates can be used as an indicator for unselective HDAC family inhibition for the two HDAC8-selective compounds. We therefore established working concentrations for the selective use of the inhibitors on neuroblastoma cells at 40 *μ*M for Cpd2 and 4 *μ*M for PCI-34051 and PCI-48000. We confirmed the HDAC8-selective inhibition under these conditions: (i) increase in whole lysine acetylation levels (Figure 1c and Supplementary Table 2), (ii) increase in acetylation level of the HDAC8 substrate core cohesion complex protein SMC3^17^ (Figure 1d), (iii) absence of HDAC6 substrate acetylation (tubulin;^18^ Figure 1d and Supplementary Figure S2) and (iv) absence of HDAC1-3 substrate acetylation (histone 4;^19^ Figure 1d and Supplementary Figure S2). Treatment with the pan-HDAC inhibitor TSA (trichostatin A), which was used as a control for unselective HDAC inhibition, resulted in a much stronger induction of global lysine acetylation (Figure 1c and Supplementary Table 2). This is in line with the higher amount of acetylated substrates as a consequence of inhibition of multiple HDACs. As predicted, increasing concentrations of Cpd2 and PCI-34051, far above their IC_50_ values, displayed HDAC6 off-target activity and induced tubulin acetylation (Figure 1d).

The treatment of a panel of MYCN-amplified as well as MYCN-single-copy neuroblastoma cell lines with HDAC8 inhibitors for 6 days significantly decreased cell numbers. Of note, medulloblastoma cell lines were less susceptible compared with neuroblastoma cells. Untransformed cells (infant fibroblasts and astrocytes) hardly responded to the treatment (Figure 1e). So far, these results point towards HDAC8 as a promising and selective target for neuroblastoma treatment.

### HDAC8-selective inhibitors display antineuroblastoma activity *in vivo*

**Figure 2:**
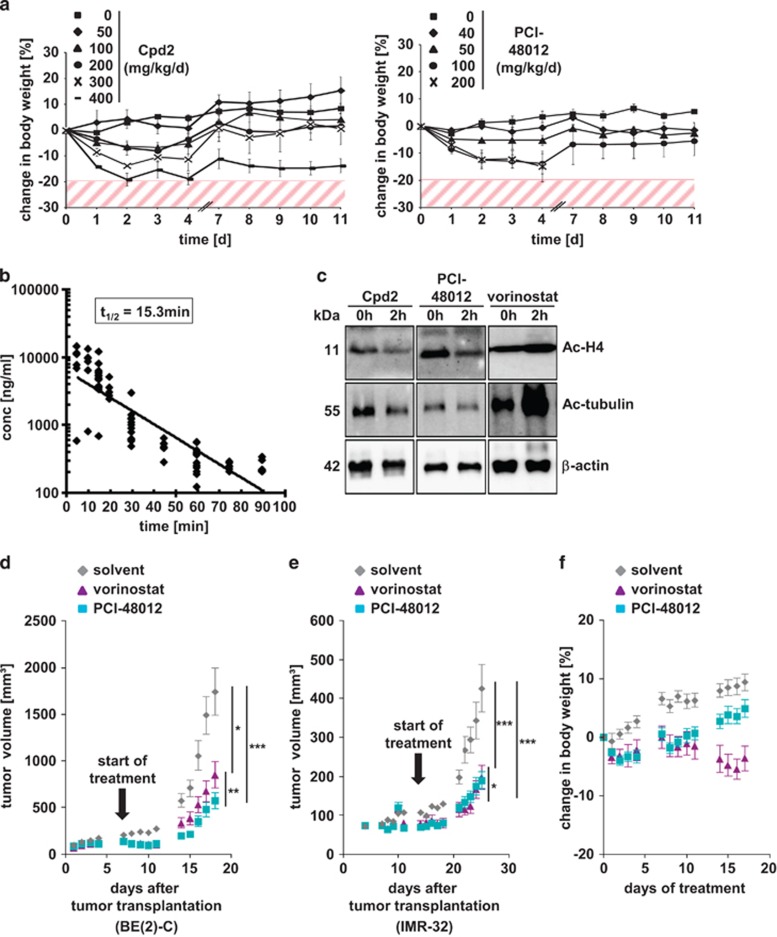
Characterization of HDAC8 inhibitors *in vivo*. (**a**) NMRI *Foxn1* nude mice were treated with different concentrations of HDAC8 inhibitors Cpd2 and PCI-48012. The DLT is defined by loss of body weight of ≥20%. Solvent control=0 mg/kg per day. Data points represent mean values and error bars represent S.E.M. (**b**) Determination of half-life of HDAC8 inhibitor Cpd2 in the blood plasma of NMRI *Foxn1* nude mice. Cpd2 was injected intraperitoneally at a dose of 100 mg/kg and whole blood was collected at several time points (0, 10, 20, 30, 45, 60, 75 and 90 min) in cohorts of six animals, respectively. Resulting concentrations were defined by HPLC analysis from blood sera. (**c**) Western blot analysis of histone 4 and *α*-tubulin acetylation levels in PBMC cells of untreated mice (0 h) and respective animals upon treatment with HDAC8 inhibitors PCI-48012 (40 mg/kg) and Cpd2 (50 mg/kg) 2 h after intraperitoneal injection. Broad-spectrum HDAC inhibitor vorinostat (100 mg/kg) was used as a positive control. Actin served as a loading control. (**d**) Growth curve of BE(2)-C neuroblastoma xenografts in nude mice treated intraperitoneally with either solvent, HDAC8 inhibitor PCI-48012 (40 mg/kg per day) or unselective HDAC inhibitor vorinostat (150 mg/kg per d) in cohorts of 12 animals each. Treatment started 7 days after implantation of tumors (black arrow). Data are represented as mean tumor volume±S.E.M. A mixed linear model with fixed slope for each group and random intercept for each mouse was used for statistical analysis (SAS ProcMixed) (*P=0.0216; **P=0.0015; ***P<0.0001). (**e**) Growth curve of IMR-32 neuroblastoma xenografts in nude mice treated intraperitoneally with either solvent, HDAC8 inhibitor PCI-48012 (40 mg/kg per day) or unselective HDAC inhibitor vorinostat (150 mg/kg per day). Treatment started 7 days after implantation of tumors (black arrow). Data are represented as mean tumor volume±S.E.M. A mixed linear model with fixed slope for each group and random intercept for each mouse was used for statistical analysis (SAS ProcMixed) (*P=0.0349; ***P<0.0001). (**f**) Changes in body weight of nude mice in a long-term treatment of 3 × 5 days with solvent, HDAC8 inhibitor PCI-48012 (40 mg/kg per day) and broad-spectrum HDAC inhibitor vorinostat (150 mg/kg per day). Data are represented as mean tumor volume±S.E.M.

To determine the *in vivo* efficacy of HDAC8-selective inhibitors in neuroblastoma, we started to characterize the inhibitors for *in vivo* use. We first determined toxicity profiles, maximum tolerable doses (MTDs) and plasma levels of the two HDAC8-selective inhibitors Cpd2 and PCI-48012 for application in mice *in vivo*. We chose the latter compound as a more *in vivo* stable variant of PCI-48000 with improved pharmacokinetic properties (Figure 1b). Both inhibitors were intraperitoneally injected into NMRI *Foxn1* nude mice in increasing doses from 40 up to 400 mg/kg per day for 2 × 5 days. Each dose was tested in a cohort of three animals. Resulting MTDs were validated within cohorts of eight animals for each inhibitor. Dose-limiting toxicities (DLTs) were determined by monitoring body weight, where a reduction of ≥20% from starting body weight indicated toxicity. Additional experiments performed to check for toxicity included clinical chemistry and hematological blood parameters, as well as histological examination of a panel of organs for toxicity using hematoxylin and eosin (H&E) stain after killing the animals. As a reference, the blood of 12 untreated NMRI *Foxn1* nude mice was analyzed. In these studies, we determined the MTD for HDAC8 inhibitors Cpd2 at 50 mg/kg per day and for PCI-48012 at 40 mg/kg per day. At these concentrations, neither body weight (Figure 2a) nor blood parameters were critically changed according to clinical toxicity criteria (Table 1). DLTs of Cpd2 included weight loss and signs of liver toxicity, as evidenced by elevated plasma liver enzymes and detection of necrotic areas on histological liver examination. DLTs of PCI-48012 included severe inflammation of the intestine at organ examination, but no noticeable changes in blood parameters. Pharmacokinetic studies after intraperitoneal administration of the inhibitors identified the half-life of Cpd2 to be ~15 min, with a plasma peak concentration of ~30 *μ*M, matching the *in vitro* HDAC8 IC_50_ of 34 *μ*M (Figure 2b). PCI-48012 has a half-life of ~1 h, with a plasma peak concentration of 8.24 *μ*M after administration of a single dose of 100 mg/kg, suggesting that the MTD of 40 mg/kg results in therapeutic plasma levels above the HDAC8 *in vitro* IC_50_, but still in the range of selectivity. As a pharmacodynamic marker for HDAC8 inhibitory action *in vivo*, we analyzed lysine acetylation levels in peripheral blood mononuclear cells (PBMC) after intraperitoneal administration of Cpd2 and PCI-48012 at MTD concentrations (Supplementary Table 2). HDAC8 selectivity was further controlled by the absence of tubulin acetylation and absence of histone 4 acetylation in PBMC (Figure 2c). Vorinostat was used as a control for unselective HDAC inhibition. The MTD of vorinostat in mice (150 mg/kg per day) is well established and was taken from the published literature.^20^

Next, we determined the *in vivo* efficacy of HDAC8-selective inhibition in neuroblastoma xenograft mouse models. Because the pharmacokinetic studies revealed a relatively short half-life for Cpd2, we subsequently used PCI-48012 for treatment efficacy studies. One week after tumor cell injection, mice established a palpable tumor mass and the animals were then randomly assigned to treatment and control cohorts. Treatment with PCI-48012 significantly delayed tumor growth compared with solvent-treated control group. Moreover, HDAC8-selective inhibition delayed neuroblastoma tumor growth more efficiently (BE(2)-C model; Figure 2d) or equally efficiently (IMR-32 model; Figure 2e) compared with vorinostat treatment. Whereas vorinostat treatment induced clear signs of toxicity, such as diarrhea and weight loss at the applied dose, HDAC8-selective inhibitor treatment was well tolerated (Figure 2f).

### HDAC8-selective inhibition promotes differentiation, delays cell proliferation and induces cell death *in vitro* and *in vivo*

**Figure 3:**
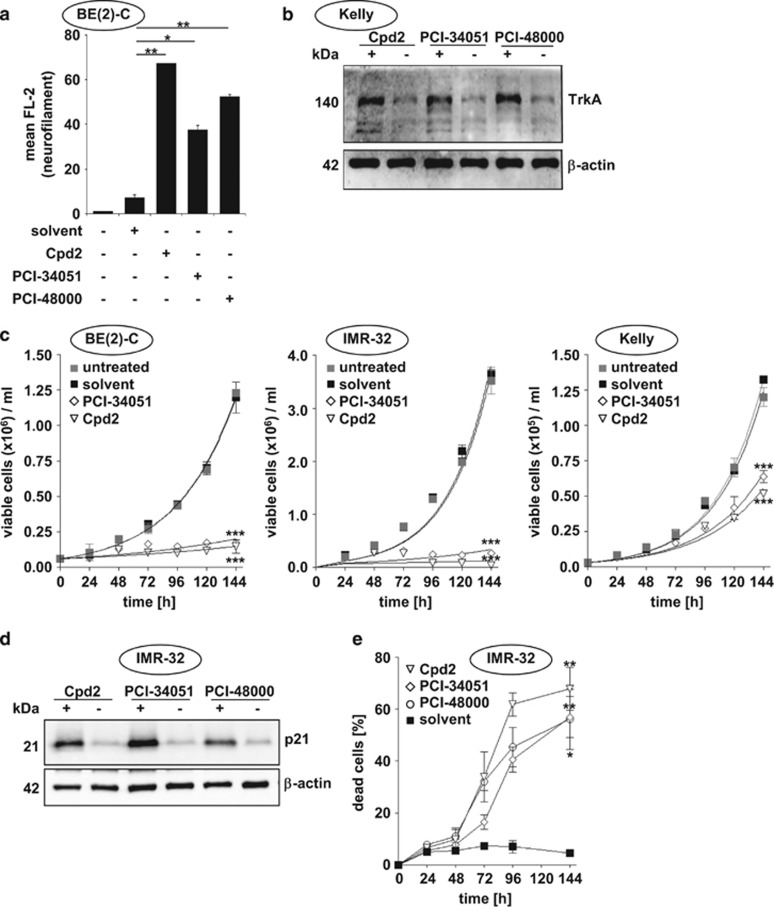
Phenotype characterization of HDAC8 inhibitors *in vitro*. (**a**) Flow cytometric quantitative analysis of neurofilament protein expression. BE(2)-C cells were treated with HDAC8 inhibitors or solvent for 6 days. Blank (–/–/–/–): secondary antibody only. (**b**) Western blot analysis of TrkA protein levels. Kelly cells were treated with HDAC8 inhibitors or solvent for 72 h. Actin served as a loading control. (**c**) Growth curves of BE(2)-C, IMR-32 and Kelly cells treated with HDAC8 inhibitors Cpd2 (40 *μ*M) and PCI-34051 (4 *μ*M). (**d**) Western blot analysis of p21^WAF1/CIP1^ protein levels. IMR-32 cells were treated with HDAC8 inhibitors (+) Cpd2 (40 *μ*M), PCI-34051 (4 *μ*M) and PCI-48000 (4 *μ*M) or solvent (−) for 72 h. Actin served as a loading control. (**e**) Time-resolved determination of dead cells via automated cell counting and trypan blue staining (% trypan blue positive) in IMR-32 cells after treatment with HDAC8 inhibitors; data points represent mean values and error bars represent S.E.M.

We have previously demonstrated that HDAC8 depletion of neuroblastoma cells via siRNA transfections resulted in an upregulation of markers for differentiation (NEF and NTRK1) as well as G0/G1 cell cycle arrest with p21^WAF1/CIP1^ upregulation. In line with our previous data, all three HDAC8 inhibitors induced morphological changes pointing towards neuronal differentiation, which was associated with an increase in neurofilament (Figure 3a) and TrkA protein expression (Figure 3b). In addition, all HDAC8 inhibitors used delayed neuroblastoma population growth (Figure 3c) and induced the cell-cycle inhibitor p21^WAF1/CIP1^ (Figure 3d). Furthermore, differentiation and proliferation disruption was followed by cell death (Figure 3e).

**Figure 4:**
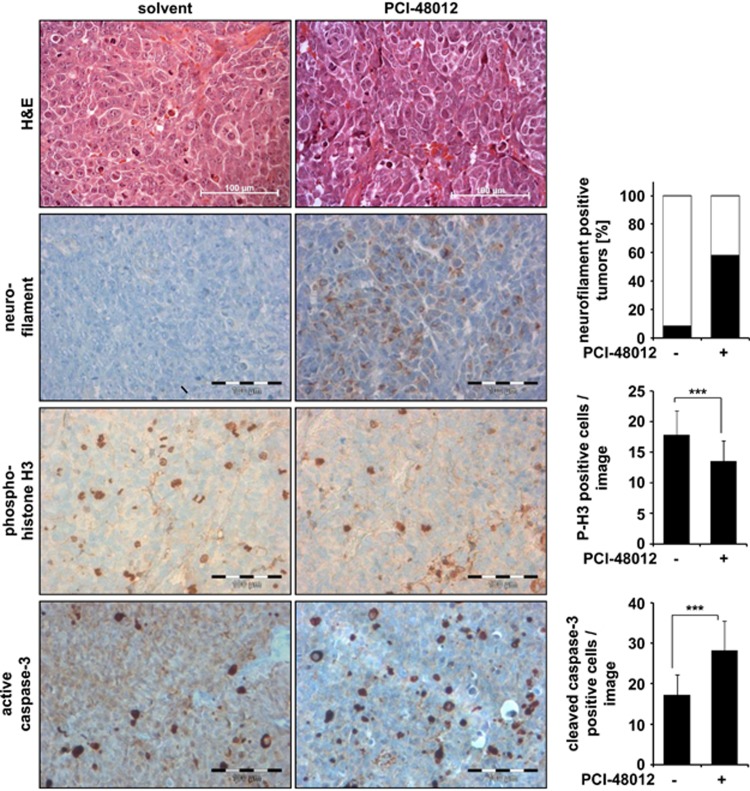
Phenotype characterization of HDAC8 inhibitors *in vivo*. Shown are representative pictures from tumors of HDAC8-inhibitor-treated animals (PCI-48012 40 mg/kg per day) and solvent-treated animals. H&E-stained sections, magnification × 400. Neurofilament (brownish color); bar chart reflects the quantitative analysis of neurofilament-positive tumors (percentage). The quantification was carried out blinded to the treatment. Phosphorylated histone H3-positive cells (brownish color); bar chart reflects the quantitative analysis of phospho-histone H3-positive cell numbers; *t*-test was used for statistics; bars represent mean values of 60 pictures (12 tumors per treatment and 5 pictures per slide) and error bars represent S.D. Active caspase-3 (brownish color); bar chart reflects the quantitative analysis of caspase-3-positive cell numbers; *t*-test was used for statistics; bars represent mean values, error bars represent S.D. Scale bars=100 *μ*m. Different regions are shown for the different stainings

Corresponding to these *in vitro* data, quantitative immunohistochemical analysis of the dissected tumor material from the HDAC8-inhibitor-treated mice revealed an increase in neurofilament-positive tumors (7/12=58.3%) compared with control groups (1/12=8.3%), and a significantly lower amount of cells positive for phosphorylated histone H3, a marker for mitosis (Figure 4).

Consistent with the cell culture results, induction of differentiation was also accompanied by induction of cell death *in vivo*. The amount of tumor cells positive for active caspase-3 was significantly increased. Taken together, selective inhibition of HDAC8 enhanced differentiation, reduced tumor growth and induced cell death *in vitro* and *in vivo*.

### Enhancement of differentiation with combined treatment of neuroblastoma cells with HDAC8 inhibitor and retinoic acid *in vitro* and *in vivo*

**Figure 5:**
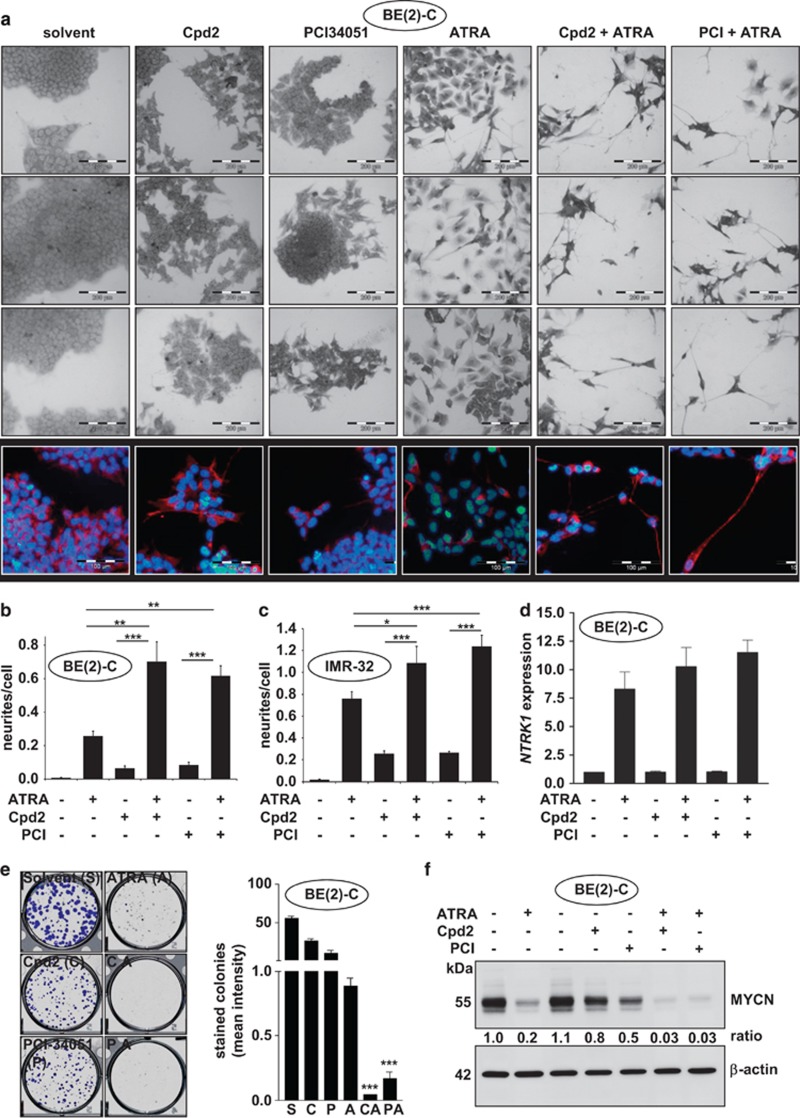
Combined treatment of neuroblastoma cells with HDAC8 inhibitors and retinoic acid *in vitro*. (**a**) Representative pictures showing crystal violet-stained BE(2)-C cells 6 days after treatment with HDAC8 inhibitors Cpd2 (20 *μ*M) or PCI-34051 (2 *μ*M) and ATRA (10 *μ*M) (scale bar=200 *μ*m). Immunofluorescent pictures show neurofilament (red) and DAPI (blue) staining of BE(2)-C cells 6 days after treatment with HDAC8 inhibitors Cpd2 (20 *μ*M) or PCI-34051 (2 *μ*M) and ATRA (10 *μ*M) (scale bar=100 *μ*m). (**b**) Bar chart reflects the quantitative analysis of the number of neurites per cell of BE(2)-C cells. The quantification was carried out blinded to the treatment. *T*-test was used for statistics (*P<0.05, **P<0.01 and ***P<0.001). Bars represent mean values and error bars represent S.D. (**c**) Bar chart reflects the quantitative analysis of the number of neurites per cell of IMR-32 cells. *T*-test was used for statistics (*P<0.05, **P<0.01 and ***P<0.001). Bars represent mean values and error bars represent S.D. (**d**) Bar diagram displays *NTRK1* mRNA expression 6 days after treatment of BE(2)-C cells with HDAC8 inhibitors Cpd2 (20 *μ*M) and PCI-48000 (1 *μ*M) in combination with ATRA (10 *μ*M). (**e**) Longer-term colony assay of BE(2)-C cells treated once with HDAC8 inhibitors Cpd2 (C; 20 *μ*M) and PCI-34051 (P; 2 *μ*M), and ATRA (A; 10 *μ*M) for at least 10 days. Results were quantified and are displayed in the bar diagram. Bars represent mean values and error bars represent S.E.M. (***P<0.001). (**f**) Western blot analysis of MYCN protein levels in BE(2)-C cells 6 days after treatment with HDAC8 inhibitors Cpd2 (20 *μ*M) or PCI-34051 (2 *μ*M) and ATRA (10 *μ*M)

Because we observed induction of differentiation following selective HDAC8 inhibition, we wondered whether it is possible to further enhance the differentiation phenotype via combination with retinoic acid, a differentiating agent currently in use for neuroblastoma therapy. In these experiments, we used the HDAC8 inhibitors in lower concentrations (Cpd2 20 *μ*M and PCI-48000 2 *μ*M), which were still sufficient to inhibit HDAC8 (Supplementary Table 2). The combined treatment increased the amount of neurite outgrowths as well as neurite length in neuroblastoma cell lines (Figures 5a–c and Supplementary Figure S3A) and increased the expression of neurofilament (Figure 5a and Supplementary Figure S3A), as well as NTRK1 (Figure 5d). Additionally, the combined treatment decreased cell numbers (Supplementary Figures S3B–D) and the ability to form colonies in longer-term assays (Figure 5e and Supplementary Figure S3E). One already well-known result of retinoic acid treatment in neuroblastoma is the reduction of MYCN oncogene expression in amplified tumors.^21^ Interestingly, the cotreatment with HDAC8-selective inhibitors even further reduced MYCN protein levels in BE(2)-C and IMR-32 cells (Figure 5f and Supplementary Figure S3F).

**Figure 6:**
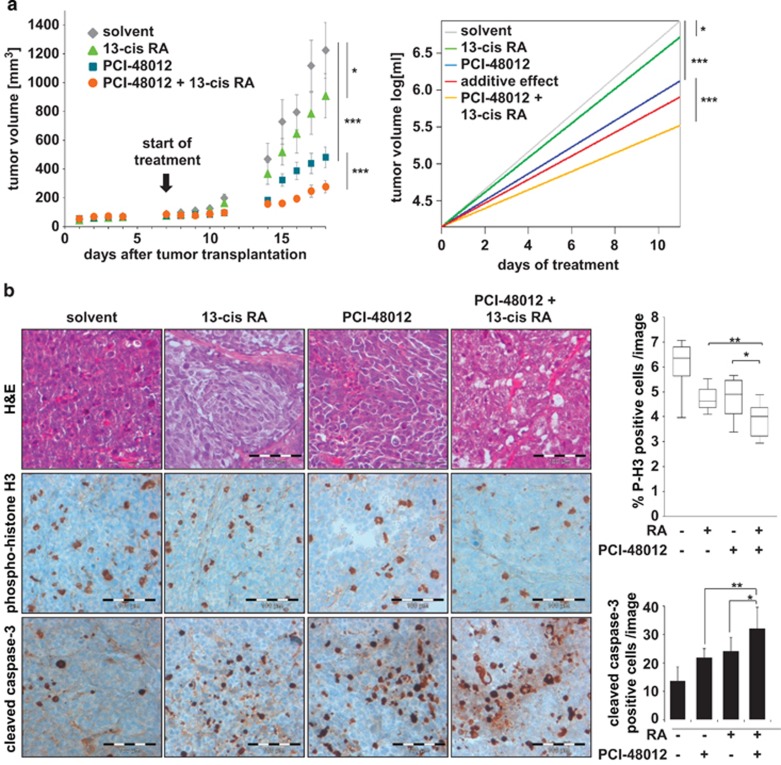
Combined treatment with HDAC8 inhibitors and retinoic acid *in vivo*. (**a**) Left panel: Growth curves of BE(2)-C neuroblastoma xenografts in NMRI *Foxn1* nude mice treated i.p. with HDAC8 inhibitor PCI-48012 (40 mg/kg/d), 13-*cis* retinoic acid (10 mg/kg/d) or combination of both compounds. Cohorts consisted of 12 animals each. Treatment started 7 days after implantation of tumors (black arrow). Data are presented as mean tumor volume±S.E.M.. Right panel: For assessment of *in vivo* effects, tumor volumes were log-transformed. A linear mixed model was used with fixed effect for slope in each group and random intercept for each mouse. Statistical analysis of additive or synergistic effects was performed using SAS Proc Mixed by evaluation of the interaction effect. The right panel shows the predicted change of log tumor volumes over time, displayed as straight lines with group-specific slope. The red line shows the hypothetical change of log tumor volume if the combinational treatment was additive. Synergistic effects produce a line below the red line. *P<0.05, ***P<0.001. (**b**) Immunohistological analysis of tumor material. Shown are representative pictures from tumors of animals treated with either HDAC8-inhibitor, 13-*cis* retinoic acid or the combination of both compounds. Hematoxylin & eosin (H&E) stained sections, magnification 400x. Phosphorylated histone H3-positive cells were counted and quantified as a percent of cells per image; *t*-test was used for statistics (*P<0.05, **P<0.01). Active caspase-3 (brownish color); bar chart reflects the quantitative analysis of caspase-3 positive cell numbers; *t*-test was used for statistics; bars represent mean values, error bars represent S.D. Scale bars=100 *μ*m. Different regions are shown for the different stainings

In addition, we combined HDAC8-selective inhibitor PCI-48012 with retinoic acid *in vivo*. For these experiments, the clinically approved drug 13-*cis* retinoic acid (13-*cis* RA) was used instead of ATRA because of its prolonged *in vivo* half-life in comparison with ATRA.^22^ We first determined a tolerable combination regimen of HDAC8 inhibitor plus retinoic acid. HDAC8 inhibitor PCI-48012 (40 mg/kg per day) was injected intraperitoenally in combination with 13-*cis* RA at different concentrations into NMRI nude mice. Toxicity of the combination was assessed by monitoring body weight (Supplementary Figure S4A). These experiments revealed a tolerable dose for 13-*cis* RA of 10 mg/kg per day, as intraperitoneal administration in a dose of 20 mg/kg per day caused severe swelling of the abdomen. Treatment of BE(2)-C xenografted NMRI nude mice for 2 × 5 days with HDAC8 inhibitor, or 13-*cis* RA revealed that the treatment with HDAC8 inhibitor alone was more potent to reduce tumor growth than 13-*cis* RA treatment alone. The combination of both agents was more potent than either treatment alone and resulted in synergistic effects, which was determined using log-transformed tumor volumes with a linear mixed model (Figure 6a and Supplementary Figure S4B). The *in vivo* combination of PCI-48012 and 13-*cis* RA decreased tumor proliferation and increased tumor cell death in comparison with either single treatment alone (Figure 6b). In summary, HDAC8-selective inhibition enhanced antineuroblastoma activity of retinoic acid treatment *in vitro* and *in vivo*.

### Forced HDAC8 overexpression counteracts retinoic acid-mediated differentiation

**Figure 7:**
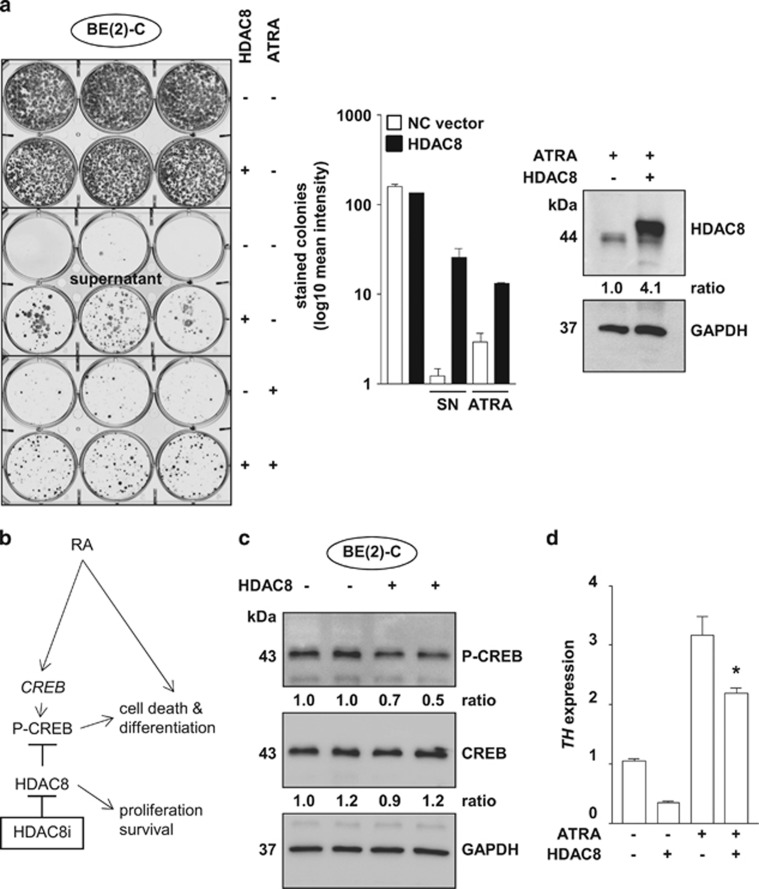
HDAC8 overexpression counteracts ATRA treatment. (**a**) Longer-term colony assay (18 days) of BE(2)-C cells stably overexpressing HDAC8, treated with ATRA (10 *μ*M), where indicated. Supernatant containing detached floating cells from densely grown untreated cells was transferred into a new 6-well plate and colonies were stained 10 days after replating. Results were quantified and are displayed in the bar diagram. Bars represent mean values and error bars represent S.E.M. (***P<0.001). SN, supernatant cells. (**b**) Hypothetical model: HDAC8 and ATRA signaling might converge at the level of CREB. Combined treatment of neuroblastoma cells with HDAC8 inhibitors and retinoic acid might enhance CREB-mediated differentiation. (**c**) Western blot analysis of BE(2)-C cells stably overexpressing HDAC8, where indicated, for P-CREB and CREB levels. (**d**) Bar diagram displays *TH* mRNA expression 72 h after treatment of BE(2)-C cells stably overexpressing HDAC8, where indicated, with ATRA (10 *μ*M) (*P<0.05).

Next, we used stably HDAC8-overexpressing BE(2)-C cells for longer-term colony assays (18 days), and treated the cells with ATRA. During this time period, untreated cells (HDAC8 and empty vector control) grew to a high density and began to detach, and thus the differences were not visible anymore. However, reseeding of detached cells floating in the supernatant into new plates revealed a substantial difference between HDAC8-overexpressing and empty vector control cells (Figure 7a, labeled as ‘supernatant'). In addition, ATRA efficiently reduced the ability of empty vector control cells to form colonies and significantly more colonies were formed by ATRA-treated HDAC8-overexpressing cells (Figure 7a), suggesting a direct mechanistic link between HDAC8 and retinoic acid signaling. With this in mind, we focused on CREB, because CREB is involved in both retinoic acid^23^ and HDAC8 signaling (Figure 7b). HDAC8 has been described to be involved in the phosphatase-mediated inactivation of CREB.^24^ Indeed, stably HDAC8-overexpressing BE(2)-C cells displayed lower levels of phosphorylated CREB (Figure 7c). In addition, expression of tyrosine hydroxylase, which has been described to be regulated by CREB via direct promoter activation (Armstrong *et al.*^25^ and references therein), was repressed in stably HDAC8-overexpressing BE(2)-C cells (Figure 7d). Thus, we hypothesize that HDAC8 and ATRA signaling partly converge at the level of CREB and the combined treatment of neuroblastoma cells with HDAC8 inhibitors and retinoic acid enhances differentiation.

## Discussion

The treatment outcome of high-risk neuroblastoma tumors still needs to be improved. Ongoing studies focus on the identification of novel therapeutic approaches aiming at oncogenic molecular targets to improve therapy efficacy for stage 4 patients.^26, 27^ The use of HDAC inhibitors is emerging as an effective treatment strategy for cancer therapy.^28, 29^ Several HDAC inhibitors are being tested in phase I–III clinical trials and show significant responses in leukemias and lymphoma. Most HDAC inhibitors block the activity of multiple HDAC isozymes involved in numerous biological processes, as demonstrated by the loss of function studies in mice. For example, the knockout of class I HDACs 1 and 3 manifests in early embryonic lethality in mice and depletion of HDAC2 results in lethal cardiac hypertrophy.^30, 31, 32^ Simultaneous inhibition of the activity of these HDACs yields a high potential for toxicities that result in dose-limiting side effects, restricting the full anticancer potential of HDAC inhibitors. Hence, inhibition of one single HDAC isozyme could be more effective and less toxic than the unspecific inhibition of several HDAC family members by creating a larger therapeutic window. This study demonstrates superiority of selective HDAC isozyme targeting *versus* pan-HDAC inhibition in terms of toxicity and efficacy in a tumor model that is dependent on HDAC8.

We chose HDAC8-selective targeting in neuroblastoma for this direct comparison since in our previous studies we observed a particular oncogenic function of HDAC8 in neuroblastoma among all HDAC family members investigated: advanced-stage and metastasized neuroblastoma tumors express high levels of HDAC8.^15^ Here, we demonstrate the antineuroblastoma efficacy of an HDAC8 inhibitor *in vivo* at concentrations avoiding unspecific and therapy-limiting side effects. Vorinostat has been reported to inhibit primarily HDACs 1, 2, 3 and 6,^33^ which are the ubiquitously expressed HDAC family members that are hypothesized to be responsible for unwanted side effects.^10^ In contrast, HDAC8 expression has been found to be rather tissue-specific.^34^ Thus, in terms of unspecific side effects, the targeting of one single enzyme seems to be superior to pan-HDAC inhibition when applied in an appropriate tumor entity that displays oncogenic dependency on that particular HDAC family member. Nevertheless, besides HDAC8, other HDAC family members also control tumor-suppressive functions in neuroblastoma. For example, HDAC1 in the sensitization of multidrug-resistant neuroblastoma cell lines to cytotoxic agents,^35^ HDAC2 in repressing miR-183-mediated tumor suppression^36^ and HDAC3 in negatively regulating tumor suppressor GRHL1.^37^ Recently, we have discovered a novel function for HDAC10 in promoting autophagy-mediated cell survival. HDAC10 depletion in neuroblastoma cells interrupted autophagic flux and induced accumulation of autophagosomes, lysosomes and a substrate of the autophagic degradation pathway, p62/SQSTM1. Disrupted autophagy was associated with sensitization to cytotoxic drug treatment in a panel of highly malignant neuroblastoma cell lines.^38^

Our experiments also focused on the understanding of the molecular function of HDAC8 in neuroblastoma to find rationally targeted treatment combinations for clinical application of HDAC8-selective inhibitors. So far, HDAC8 has been reported to have specific function in the development of cranial neural crest cells of mice, as knockout of HDAC8 leads to skull instability and perinatal death.^39^ Recently, human HDAC8 variants have been described, which are associated with X-linked intellectual disability disorders. All males that carried the variant in HDAC8 showed microcephaly and distinct focal deformation of the skull.^39^ HDAC8 itself has been described to be regulated by SOX4,^40^ a transcription factor of the SOX family, which is required for B-lymphocyte development,^41^ and also during development of the sympathetic nervous system.^42, 43^

The identification of HDAC8-specific substrates is still of major interest and would be the best tool for HDAC8 inhibitor on-target validation in tumor tissue. Quite recently, the cohesion complex protein SMC3^17^ and the tumor suppressor ARID1A^44^ have been described to be deacetylated in an HDAC8-dependent manner. Additionally, HDAC8 has been linked to phosphatase PP1-mediated CREB inactivation.^24^ Activation of CREB during ATRA treatment has been described before^45^ and phosphorylation of CREB has been postulated to mediate retinoic acid-induced caspase-8 expression in neuroblastoma cells.^23^ Caspase-8 is frequently inactivated by epigenetic silencing in many tumors, including neuroblastomas, and the expression was induced by the use of epigenetically acting compounds in combination with interferon-γ.^46^ As RA treatment has been described to activate CREB and HDAC8 expression inactivates CREB, we hypothesize that the combined treatment with RA and HDAC8 inhibitors might synergistically act on CREB-mediated cell differentiation. Indeed, our data show that a combination of both HDAC8 inhibitor and 13-*cis* retinoic acid, a currently applied drug in neuroblastoma treatment protocols, strongly enhanced differentiation in cell culture and decreased tumor growth *in vivo*. Mechanistically, HDAC8 counteracts retinoic acid-mediated CREB signaling and inhibition of clonogenic growth and combined treatment with HDAC8 inhibitor and ATRA enhance CREB phosphorylation. This suggests that the molecular action of both compounds converge at the level of CREB. However, the effects on CREB phosphorylation were rather modest and further experiments are required to unravel completely the link between retinoic acid and HDAC8 inhibitor combination treatment-induced cell death and differentiation mechanism in neuroblastoma.

Taken together, our data demonstrate effectiveness of an HDAC-selective inhibitor in a preclinical model of neuroblastoma. From a clinical perspective, combination of HDAC8 inhibition with retinoic acid treatment might be a promising strategy in the maintenance treatment of high-risk neuroblastoma.

## Materials and Methods

### Cell culture and transfections

All cell lines were grown under standard conditions as described previously.^38^ Human neuroblastoma cell lines BE(2)-C (ECACC, Salisbury, UK), IMR-32 (DSMZ, Braunschweig, Germany), Kelly (DSMZ), SH-SY5Y (DSMZ), SK-N-AS and SH-EP (both generously provided by the laboratory of M Schwab), and medulloblastoma cell lines UW-228-2, DAOY and ONS76^47^ were grown under standard conditions in DMEM with l-glutamine, 4.5 g/l glucose (Lonza, Basel, Switzerland) and 1% non-essential amino acids (NEAA) (Invitrogen, Darmstadt, Germany), or RPMI1640 with l-glutamine (Lonza) and 1% NEAA, or EMEM with l-glutamine. All media were supplemented with 10% fetal bovine serum (FBS) (Sigma, Munich, Germany). Non-transformed, proliferatively active primary human skin fibroblasts from an infant donor were a friendly gift from Petra Boukamp, German Cancer Research Center (DKFZ), Heidelberg, Germany. Fibroblasts were maintained in DMEM/HAM's F12 (Invitrogen), supplemented with 10% FBS and 1% NEAA. Neuroblastoma cell lines were last genotyped in April 2012 (DSMZ). Medulloblastoma cell lines were last genotyped in October 2012 (DKFZ). All cell lines were routinely tested for mycoplasma contamination. Human infant astrocytes (obtained from S Pfister, German Cancer Research Center (DKFZ)) were grown as described previously.^38^

Transient transfections were performed as described previously.^15^ The following siRNAs were used: HDAC8 (siRNA1 (ID 120597, exons 1 and 2; Ambion (Huntingdon, UK) Ltd)) and corresponding negative control siRNA (Silencer Negative Control 1; Ambion).

#### Generation of stable cell lines

Human BE(2)-C cell lines stably expressing HDAC8 were established by transfection using Effectene (Qiagen, Hilden, Germany) with pCEP4/hygro-FLAG HDAC8 cDNA or hygromycin-resistant empty vector control (pCEP4/hygro-FLAG). Transfected cells were selected with hygromycin (400 *μ*g/ml) for 3 weeks. A mixed population of hygromycin-resistant cells was used for experimental analysis.

### Animal studies

#### Mouse xenograft studies with HDAC8 knockdown

BE(2)-C cells transiently transfected with siRNA against HDAC8 or negative control were resuspended in Matrigel (BD, Franklin Lakes, NJ, USA) and 20 U/ml heparin 48 h after transfection. Next, 1.3 × 10^6^ viable cells were implanted in 100 *μ*l Matrigel into the subcutaneous tissue of right flank of 5- to 6-week-old female athymic nude mice (NMRI *Foxn1*; Harlan Laboratories Inc., Nijmegen, The Netherlands) in cohorts of 20 animals each. Cell numbers were established via trypan blue exclusion. Tumor dimensions were measured and tumor volume (mm^3^) was calculated as (*L* × *W*^2^) × *π*/6 (*L* indicating length, *W* indicating width (all in mm)). At days 3, 6 and 9 after tumor cell injection, three randomly selected mice from each group were killed, and tumors were explanted. Specimens used for isolation of total RNA or protein were shock frozen in liquid nitrogen immediately after removal and stored at −80 °C. A part of each tumor sample was formalin fixed and embedded in paraffin for immunohistochemical analysis. The transfection efficiency of siRNA in BE(2)-C cells was 90% as determined by fluorescently labeled siRNA siGLO Lamin A/C (Dharmacon, Lafayette, CO, USA).

#### Preparation of PBMC cells

Whole blood (100 *μ*l) was collected from the femoral vein of untreated athymic NMRI nude mice (HsdCpb:NMRI-Foxn1^nu^; Harlan Laboratories Inc.), and respective animals were killed 2 h after intraperitoneal injection of substances. For the analysis of whole acetylation levels, mice were killed at half-life time of the inhibitors. Whole blood with heparin was added to RBC lysis buffer (diluted 1 : 10) (BioLegend, San Diego, CA, USA) at a ratio of 1 : 4. Next, red blood cell lysis was performed according to the manufacturer's instructions. PBMCs were lysed in SDS lysis buffer for western blot or processed for FACS analysis.

#### Determination of inhibitor plasma half-life

To determine the half-life of HDAC8 inhibitor Cpd2 *in vivo*, the substance was injected intraperitoneally at a dose of 100 mg/kg into athymic NMRI nude mice (Harlan). Whole blood (100 *μ*l) was collected from the femoral vein of animals at time points 0, 5, 10, 15, 20, 30, 45, 60, 75 and 90 min after injection (six animals per time point). Plasma was obtained by centrifugation at 10 000 r.c.f. for 10 min at 4 °C and stored at −80 °C. Plasma samples (50 *μ*l) were analyzed for Cpd2 concentration after liquid/liquid extraction with tert.-butylmethylether using HPLC coupled to tandem mass spectrometry (LC/MS/MS; TSP 2000, TSQ 7000; Thermo Fisher Scientific, Waltham, MA, USA). The extracts were gradient chromatographed on a Hydro-RP column (4 *μ*M, 150 × 2.1 mm^2^; Phenomenex, Torrance, CA, USA) at 40 °C using 5 mM ammonium acetate and acetonitrile at 0.6 ml/min. The eluent was directly infused into the electrospray ion source (negative ions) of the MS/MS system and the mass transitions of Cpd2 (*m*/z 186.1→142.0 and *m/z* 186→58.0) and vorinostat (internal standard; *m/z* 268.1→97.0) were monitored in the multiple reaction monitoring mode. Cpd2 and vorinostat eluted within 6 min and the respective peak area ratios were used for the quantification of Cpd2 using linear regression in the calibrated range from 100 to 20 000 ng/ml. The limit of quantification was 100 ng/ml and the correlation coefficient was >0.99. Accuracy and precision control using quality control samples revealed deviation consistently below 15%, which is in accordance with the FDA guideline, *Guidance for Industry: Bioanalytical Method Validation.* All animal studies were approved by the German Cancer Research Center (DKFZ) institutional animal care and use committee and the Regional Administrative Council Karlsruhe, Germany. All experiments were in accordance with the relevant regulatory standards.

#### Determination of MTD

HDAC8-selective inhibitors were intraperitoneally injected into athymic NMRI nude mice (Harlan) in increasing doses from 40  to 400 mg/kg per day. DLTs were monitored by observation of general condition and determination of body weight. We used several criteria for objectively measuring DLT. These included a decrease in body weight of ≥20% compared with baseline body weight, blood analysis of standard parameters for clinical chemistry and hematology (central laboratory of University Hospital Heidelberg, Heidelberg, Germany) and organ histopathology (formalin-fixed, paraffin-embedded organs), by H&E stain for the detection of necrosis (Prof. Dr. AD Gruber, Institute for Veterinary Pathology, Berlin, Germany). These measurements assured lack of toxicities at MTD concentrations of inhibitors.

#### Mouse xenograft studies with HDAC8 inhibitors

Neuroblastoma cells were resuspended in Matrigel and 20 U/ml heparin. Next, 2 × 10^6^ (BE(2)-C) or 4 × 10^6^ (IMR-32) viable cells were implanted in 100 *μ*l Matrigel into the subcutaneous tissue of right flank of 5- to 6-week old female athymic nude mice (Harlan). Mice were randomly assigned to groups of 12 individuals bearing similarly sized tumors. HDAC8-selective inhibitor PCI-48012 at MTD (40 mg/kg per day) was dissolved in 90% DMSO (HybriMax; Sigma), 10% PBS and given by intraperitoneal injection for 2 × 5 days. HDAC inhibitor vorinostat was dissolved in 100% DMSO and given by intraperitoneal injection at a concentration of 150 mg/kg per day for 2 × 5 days. 13-*Cis* retinoic acid (Sigma) was dissolved in 90% DMSO, 10% PBS and given by intraperitoneal injection at a concentration of 10 mg/kg per day for 2 × 5 days. Tumor volume (mm^3^) was calculated as described above (*L* × *W* × *H*) × *π*/6, where *L* indicates length, *W* indicates width and *H* indicates height (all in mm)). At explantation, tumor material was processed as described above.

All animal studies were approved by the German Cancer Research Center (DKFZ) institutional animal care and use committee and the Regional Administrative Council Karlsruhe, Germany. All experiments were in accordance with the relevant regulatory standards.

### Reagents

HDAC8-selective inhibitors Cpd2^13^ (stock concentration 250 mM), PCI-34051 (Figure 2a)^14^ (stock 20 mM; Pharmacyclics Inc., Sunnyvale, CA, USA), PCI-48000 (stock 20 mM; Pharmacyclics) and PCI-48012 (Pharmacyclics), as well as unselective HDAC inhibitors vorinostat (SAHA: suberoylanilide hydroxamic acid) (stock 100 mM; Selleck Chemicals, Houston, TX, USA) and TSA (stock 1 mM; Merck, Darmstadt, Germany), were dissolved in DMSO. Cpd2, PCI-34051, and PCI-48000 were used in *in vitro* experiments. For *in vivo* experiments, PCI-48012 was applied. PCI-48000 and PCI-48012 are chemical modifications of PCI-34051 with similar HDAC8 selectivity profiles and similar IC_50_ values, but longer plasma half-life in the case of PCI-48012. All-*trans* retinoic acid (stock 10 mM; Sigma) was dissolved in ethanol.

### *In vitro* HDAC activity assay

The inhibitory activity of HDAC8-selective inhibitors towards all classical 1–11 HDACs was investigated by Reaction Biology Corporation (Malvern, PA, USA). The catalytic domains of human HDACs were expressed by baculovirus expression system in Sf9 cells. Enzymes were stored in 50 mM Tris-HCl (pH8.0), 138 mM NaCl, 20 mM glutathione and 10% glycerol, and were stable for >6 months at −80 °C. The purity was ascertained by SDS-PAGE. Peptide substrate was conjugated with AMC. Reaction buffer was 25 mM Tris-Cl, pH8.0, 137 mM NaCl, 2.7 mM KCl, 1 mM MgCl_2_ and 0.1 mg/ml BSA. The HDAC reaction was performed at 30 °C for 2 h before adding the developer reagent. The free AMC was detected with excitation of 360 nm and emission 460 nm at kinetic mode for 90 min. IC_50_ values were calculated with GraphPad Prism version 3.0a (GraphPad Software Inc., San Diego, CA, USA).

### Cell counting, cell viability, cell death and colony assay

Cells were collected, pooled with corresponding supernatant, centrifuged and resuspended in 1.5 ml cell culture media. Cell count as well as cell viability was measured by automated trypan blue staining with Vi-Cell XR Cell Viability Analyzer from Beckman Coulter (Krefeld, Germany). Caspase-3-like protease activity assay was performed as described previously.^15^

### Colony assay

In six-well plates, 500 cells were seeded and treated as indicated. Viable colonies were stained after a minimum of 10 days with crystal violet. For quantification, the mean intensity of each well of the 8-bit binary picture was measured with ImageJ software (U. S. National Institutes of Health, Bethesda, MD, USA; http://imagej.nih.gov/ij/).

### Real-time, reverse-transcription polymerase chain reaction

Real-time PCR was performed as described previously.^15^ Data were normalized against housekeeping genes *SDHA* and *HPRT* ^48^ and set in relation to negative control.

### Western blot analysis

Western blot analysis was performed as described previously.^15^ For detection of phosphoproteins, PhosStop (Roche, Penzberg, Germany) was added to the lysis buffer. The following antibodies were used for detection: anti-HDAC8 (H-145) (polyclonal; Santa Cruz, Santa Cruz, CA, USA), anti-acetyl tubulin (clone 6-11B-1; Sigma), anti-acetyl-histone H4 (polyclonal; Upstate, Lake Placid, NY, USA), anti-acetyl-SMC3 (provided by Prof. K Shirahige, University of Tokyo, Tokyo, Japan), anti p21^waf1/cip1^ (clone CP74; Merck), anti-Trk (C-14) (polyclonal; Santa Cruz), anti-neurofilament-M (polyclonal; Merck), anti-β-actin (clone AC-15; Sigma), anti-P-CREB (Ser133; Cell Signaling, Danvers, MA, USA), anti-CREB (48H2; Cell Signaling) and anti-GAPDH (clone 6C5; Merck).

### Intracellular protein quantification via FACS

Cells were collected, washed with PBS and fixed with 2% paraformaldehyde for 15 min at room temperature (RT). Cells were permeabilized by incubation with 50 *μ*g/ml digitonin for 6 min at RT. Cells were incubated with primary antibody overnight at 4 °C. After staining with Cy3-conjugated anti-rabbit antibody, cells were analyzed by FACS using CellQuest software (BD Biosciences, San Jose, CA, USA). The following primary antibodies were used: anti-acetylated lysine (polyclonal; Cell Signaling) and anti-neurofilament-M (clone EP2460; Epitomics, Burlingame, CA, USA).

### Crystal violet staining

Cells were fixed with 2% paraformaldehyde for 15 min at RT. After washing with PBS, sterile filtered crystal violet (0.005% in H_2_O) was added for 1 h at RT. Cells were washed three times with H_2_O. Morphometrical studies were performed on the photomicroscope CKX41 (Olympus, Hamburg, Germany) and analyzed with cellB software (Olympus).

### Immunohistochemistry

Immunohistochemical stainings were performed semiautomatically on 5-*μ*M-thick sections of formalin-fixed, paraffin-embedded specimens using VENTANA Benchmark XT (Strasbourg, France). The following antibodies were applied according to the manufacturer's protocols: anti-neurofilament protein (Clone2F11; DakoCytomation, Hamburg, Germany; diluted 1 : 200), anti-phospho-histone H3 (Ser10) (polyclonal; Biocare Medical, Concord, CA, USA; diluted 1 : 100), anti-active caspase-3 (polyclonal; Abcam, Cambridge, UK; diluted 1 : 100). Antigen retrieval was performed before primary antibody exposure. Primary antibody binding sites were visualized using the Ultra-View Universal DAB Detection Kit (Ventana/Roche, Mannheim, Germany). Nuclei were automatically counterstained by hematoxylin and blueing reagent (Ventana). From each specimen, five representative digital images of neurofilament, phosphohistone H3- and caspase-3-stained sections were captured at × 200 magnification (high power field, HPF) using a photomicroscope (CKX41; Olympus). Cell imaging software cellB (Olympus) was used for the acquisition of microscopic images. The quantification was performed by operators who were blinded to the treatment group represented in the images. Positive cells were counted with the counting tool of the cellB software (Olympus). Hematoxylin and eosin (H&E) staining was analyzed at × 400 magnification (HPF) using photomicroscope Axioskop 2 (Zeiss, Oberkochen, Germany) equipped with AxioCam ICc1 (Zeiss) and AxioVision Software Release 4.6 (Zeiss).

### Statistical analysis

For *in vitro* experiments, either a two-sided *t*-test or an one-way analysis of variance was carried out (SigmaPlot 12.0). Means are represented in bar charts, and error bars represent the S.D. of at least three independent experiments. *P*-values <0.05 were considered significant. For assessment of *in vivo* effects, tumor volumes were log-transformed. A linear mixed model was used with fixed slope for each group and random intercept for each mouse. Statistical analysis of additive or synergistic effects was performed using SAS Proc Mixed (Version 9.2; SAS Institute, Cary, NC, USA). Tumor weights were analyzed with the nonparametric Mann–Whitney test (GraphPad Prism version 3.0a). Error bars represent the S.E.M. *P*-values <0.05 were considered significant.

#### Web-based gene expression analysis

R2 (R2: microarray analysis and visualization platform; http://r2.amc.nl) was used to investigate HDAC8 expression in a publically available cohort of primary neuroblastoma patients (Academic Medical Center (AMC) cohort; Gene Expression Omnibus (GEO) database accession no. GSE16476). The following probeset was used to detect HDAC8 expression: 223345_at. Patient characteristics were published previously.^16^

## Figures and Tables

**Table 1:** Mean indicated values of blood parameters from HDAC8-inhibitor-treated NMRI *Foxn1* nude mice (±S.D.) at MTD concentrations in comparison with normal range

	**Solvent (*n*=8)**	**Cpd2 (*n*=8)**	**PCI-48012 (*n*=8)**	**Normal range (*n*=12)**
Sodium	133.1±8.9	136.8±5.3	138.9±5.3	123.4–135.7 mmol/l
Potassium	5.8±0.9	6.9±2.2	6.4±2.2	3.8–6.4 mmol/l
Calcium	2.3±0.1	2.4±0.4	2.4±0.2	2.1–2.4 mmol/l
Chloride	101.9±3.9	99.9±2.2	102.0±3.7	97.3–109.7 mmol/l
Creatinine	0.2±.04	0.2±0.04	0.2±0.02	0.1–0.3 mmol/l
Phosphate	2.6±0.5	1.4±1.0	1.9±1.2	2.0–2.9 U/l
Urea	26.3±4.2	23.5±6.4	30.0±7.2	17.4–38.3 mg/dl
Uric acid	0.8±0.8	0.9±1.0	0.7±0.3	0.4–3.2 mg/dl
Glucose	203.9±28.0	187.8±39.1	174.0±18.6	176.8–238.6 mg/dl
GOT/AST	110.0±5.2	100.3±46.9	167.5±93.0	68.3–308.7 mg/dl
GPT/ALT	66.4±30.1	66.5±10.9	127.3±73.3	29.8–143.8 mg/dl
AP	98.6±26.5	123.8±35.8	81.8±33.7	62.5–117.6 U/l
GGT	−3.0±1.8	−2.0±1.0	0.5±0.5	−1.9 to 1.0 U/l
Bilirubin total	<0.2	<0.2	<0.2	<0.2 U/l
Leukocytes	3.0±1.0	4.4±1.8	4.5±1.3	1.8–6.1/nl
Erythrocytes	7.6±0.3	7.6±0.3	7.0±0.9	5.5–8.2/pl
Thrombocytes	641.0±61.4	682.0±151.1	690.9±253.1	242.5–806.8/nl
Hemoglobin	12.6±0.7	12.4±0.7	11.6±1.6	9.4–12.7 g/dl
Hematocrit	0.4±0.02	0.4±0.01	0.4±0.04	0.3–0.4 l/l

Abbreviations: AP, alkaline phosphatase; GGT, gamma-glutamyltransferase (gammaGT); GOT, glutamic oxaloacetic transaminase; GPT, glutamic pyruvic transaminase

## Abbreviations

13-cis RA: 13-cis retinoic acid
Ac-H4: acetylated histone 4
Ac-SMC3: acetylated structural maintenance of chromosomes 3 protein
Ac-tubulin: acetylated tubulin
AMC: Academic Medical Center
AP: alkaline phosphatase
ATRA: all-trans retinoic acid
Cpd2: compound 2 (1-naphthohydroxamic acid)
CREB: cAMP-response element-binding protein
GEO: Gene Expression Omnibus
GGT: gamma-glutamyltransferase
GPT: glutamic pyruvic transaminase
HDAC: histone deacetylase
HDAC8: HDAC family member 8
H&E: hematoxylin and eosin
INSS: International Neuroblastoma Staging System
MTD: maximum tolerable dose
NEF: neurofilament
NTRK1: neurotrophic tyrosine kinase receptor type 1
OT: glutamic oxaloacetic transaminase
PBMC: peripheral blood mononuclear cells
P-H3: phosphorylated histone 3
SAHA: suberoylanilide hydroxamic acid
TrkA: tropomyosin receptor kinase A
TSA: trichostatin A
