# Supplementary material for: Selective inhibition of HDAC8 decreases neuroblastoma growth in vitro and in vivo and enhances retinoic acid-mediated differentiation
Source: Cell Death Dis. 2015 Feb 19;6(2):e1657–. doi: 10.1038/cddis.2015.24 (PMC4669789; doi:10.1038/cddis.2015.24)
Supplement: Supplementary Information [file cddis201524x1.doc]

**Supplementary Material**

**Selective inhibition of HDAC8 decreases neuroblastoma growth *in vitro* and *in vivo* and enhances retinoic acid mediated differentiation**

Running title: “HDAC8 impairs cell death and differentiation”

Inga Rettig^1^, Emily Koeneke^1^, Franziska Trippel^2^, Wolf C. Mueller^3^, Jürgen Burhenne^4^, Annette Kopp-Schneider^5^, Johannes Fabian^1^, Andreas Schober^6^, Uta Fernekorn^6^, Andreas von Deimling^7^, Hedwig E. Deubzer^1,8^, Till Milde^1,8^, Olaf Witt^1,8^ and Ina Oehme^1*^

^1^Clinical Cooperation Unit Pediatric Oncology, German Cancer Research Center (DKFZ), Heidelberg, Germany; ^2^Department of Pediatric Surgery, Dr. von Hauner Children's Hospital, Ludwig-Maximilians-University Munich, Munich, Germany; ^3^Department of Neuropathology, Institute for Pathology, University of Leipzig, Leipzig, Germany; ^4^Department of Clinical Pharmacology and Pharmacoepidemiology, University Hospital Heidelberg, Heidelberg, Germany; ^5^Department of Biostatistics, German Cancer Research Center (DKFZ), Heidelberg, Germany; ^6^Ilmenau University of Technology, Institute for Micro- and Nanotechnologies, Ilmenau, Germany; ^7^Department of Neuropathology, Institute of Pathology, Ruprecht-Karls-University Heidelberg, and Clinical Cooperation Unit Neuropathology, German Cancer Research Center (DKFZ), Heidelberg, Germany; ^8^Dpt of Pediatric Oncology, Hematology and Immunology, University Hospital Heidelberg, Heidelberg, Germany.

**Figure Legends**

**Suppl. Fig. 1:** ***In vivo* validation of HDAC8 as a drug target.** **A** Analysis of *HDAC8* expression in neuroblastoma samples (AMC cohort) reveals significantly different expression among all International Neuroblastoma Staging System (INSS) stages (ANOVA p<0.001). **B** *HDAC8* tumor expression separates treatment outcome of neuroblastoma patients. Kaplan–Meier curves are shown for overall survival in neuroblastoma patients (AMC cohort) whose tumors expressed low (n = 55) or high (n = 33) levels of *HDAC8*. The average was used as the cutoff to separate the cohort into two groups. The R2 microarray analysis and visualization platform (http://r2.amc.nl) was used for calculations and is the source of the data. **C** Western Blot analysis of HDAC8 protein levels of neuroblastoma cell lines (MYCN amplified: BE(2)-C, IMR-32, Kelly and non-amplified: SH-SY5Y, SH-EP, SK-N-AS), medulloblastoma cell lines (DAOY, UW-228-2) and untransformed cells (fibroblasts). GAPDH served as a loading control. Numbers indicate the ratio of HDAC8 to GAPDH expression. **D** Relative mRNA expression of *HDAC8* in tumors transiently transfected with HDAC8 or negative control (NC) siRNA at days 0, 3, 6 and 9 after tumor transplantation.

**Suppl. Fig. 2: Validation of selective HDAC8 inhibition.** Western Blot analysis of histone 4 acetylation and alpha-tubulin acetylation. BE(2)-C cells were treated with HDAC8 inhibitors (40 µM Cpd2, 4µM PCI-34051, 4µM PCI-48000) for 6h. Pan HDAC inhibitor TSA (150nM) was used as a positive control and actin served as a loading control.

**Suppl. Fig. 3: Combined treatment of neuroblastoma cells with HDAC8 inhibitors and retinoic acid *in vitro*.** **A** Representative pictures showing crystal violet stained IMR-32 cells 6d after treatment with HDAC8 inhibitors Cpd2 (20µM) or PCI-34051 (2µM) and ATRA (10µM) (scale bar = 200µm). Immunofluorescent pictures show neurofilament (red) and DAPI (blue) staining of IMR-32 cells 6d after treatment with HDAC8 inhibitors Cpd2 (20µM) or PCI-34051 (2µM) and ATRA (10µM) (scale bar = 100µm). **B-D** Representative pictures showing crystal violet stained Kelly (**B**), SH-EP (**C**) and SK-N-AS (**D**) cells 6d after treatment with HDAC8 inhibitors Cpd2 (20µM) or PCI-34051 (2µM) and ATRA (10µM) (scale bar = 200µm). **E** Longer-term colony assay of BE(2)-C (BE), IMR-32 (IM), Kelly (Ke), SH-SY5Y (SY) and SH-EP (SH) cells treated with PCI-34051 and ATRA were indicated. After 72h of treatment, the medium was exchanged to compound-free medium. Colonies formed after 14d were quantified and are displayed in the bar diagram. Bars represent mean values, error bars represent SEM. Asterisks indicate the level of significance between testing groups (ATRA versus PCI34051/ATRA combination) from an unpaired two-tailed t test. *p<0.05; ***p<0.001. **F** Western Blot analysis of MYCN protein levels in IMR-32 cells 6d after treatment with HDAC8 inhibitor PCI-34051 (2µM), ATRA (10µM) or the combination. Actin served as a loading control. Numbers indicate the ratio of MYCN to β-actin expression.

**Suppl. Fig. S4: Combination of HDAC8-inhibitor with the clinically applied drug 13-cis retinoic acid *in vivo*.** **A** Dose determination study for combination of HDAC8-inhibitor PCI-48012 (40mg/kg/d) with 13-cis retinoic acid. Dose limiting toxicity (DLT) is defined by a ≥ 20% loss of body weight. Combinations revealed MTD of 10mg/kg/d for 13-cis retinoic acid, as a dose of 20mg/kg/d resulted in severe swelling of the abdomen. Each concentration was tested in cohorts of three animals; error bars represent SD. **B** Shown are explanted tumors of animals treated with HDAC8-inhibitor, 13-cis retinoic acid or combination of both compounds. Scale bar = 1cm.
