# Supplementary material for: Selective inhibition of HDAC8 decreases neuroblastoma growth in vitro and in vivo and enhances retinoic acid-mediated differentiation
Source: Cell Death Dis. 2015 Feb 19;6(2):e1657–. doi: 10.1038/cddis.2015.24 (PMC4669789; doi:10.1038/cddis.2015.24)
Supplement: Supplementary Table S1 [file cddis201524x2.doc]

**Supplementary Table 1.**

IC_50_ values [µM] of HDAC8 inhibitors

|  | Cpd2 | PCI-34051 |
| --- | --- | --- |
| HDAC1 | * | 59.0 |
| HDAC2 | * | * |
| HDAC3 | * | * |
| HDAC4 | * | * |
| HDAC5 | * | * |
| HDAC6 | 75.6 | 30.0 |
| HDAC7 | * | * |
| HDAC8 | 33.7 | 0.017 |
| HDAC9 | * | * |
| HDAC10 | * | * |
| HDAC11 | * | * |
| * The calculated IC_50_ value of this HDAC is  above the highest concentration included  in the assay | | |
|
