# Supplementary material for: Selective inhibition of HDAC8 decreases neuroblastoma growth in vitro and in vivo and enhances retinoic acid-mediated differentiation
Source: Cell Death Dis. 2015 Feb 19;6(2):e1657–. doi: 10.1038/cddis.2015.24 (PMC4669789; doi:10.1038/cddis.2015.24)
Supplement: Supplementary Table S2 [file cddis201524x3.doc]

**Supplementary Table 2**

Changes in whole acetyl-lysine mean fluorescent values upon treatment with HDAC8 inhibitors (Blank-corrected)

|  |  | |  |  | | |  |
| --- | --- | --- | --- | --- | --- | --- | --- |
| Treatment | | Mean +/- SD | | | | | |
| **neuroblastoma cell culture** | |  | | |  |  | |
| solvent | | 24.7 | | | ± | 0.9 | |
| Cpd2 (40µM) | | 36.9 | | | ± | 1.3 | |
| PCI-48000 (4µM) | | 31.6 | | | ± | 1.8 | |
| TSA (150nM) | | 115.1 | | | ± | 22.0 | |
|  | |  | | |  |  | |
|  | |  | | |  |  | |
| solvent | | 34.4 | | | ± | 8.4 | |
| Cpd2 (20µM) | | 56.9 | | | ± | 7.1 | |
| PCI-48000 (1µM) | | 51.0 | | | ± | 3.4 | |
| TSA (75nM) | | 92.7 | | | ± | 5.1 | |
|  | |  | | |  |  | |
| **PBMCs** | |  | | |  |  | |
| solvent | | 32.1 | | | ± | 2.7 | |
| Cpd2 (50 mg/kg/d) | | 39.2 | | | ± | 4.3 | |
| PCI-48012 (40 mg/kg/d) | | 39.7 | | | ± | 2.5 | |
| vorinostat (150 mg/kg/d) | | 54.0 | | | ± | 4.2 | |
|  | |  | | |  |  | |
