# Supplementary figures and images for: Selective inhibition of HDAC8 decreases neuroblastoma growth in vitro and in vivo and enhances retinoic acid-mediated differentiation
Source: Cell Death Dis. 2015 Feb 19;6(2):e1657–. doi: 10.1038/cddis.2015.24 (PMC4669789; doi:10.1038/cddis.2015.24)

**A**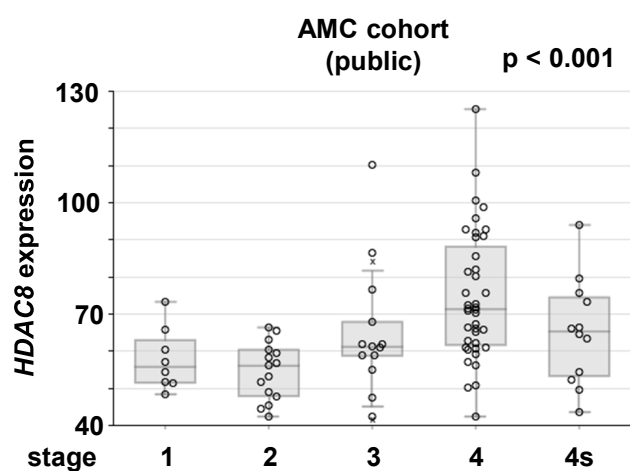**B**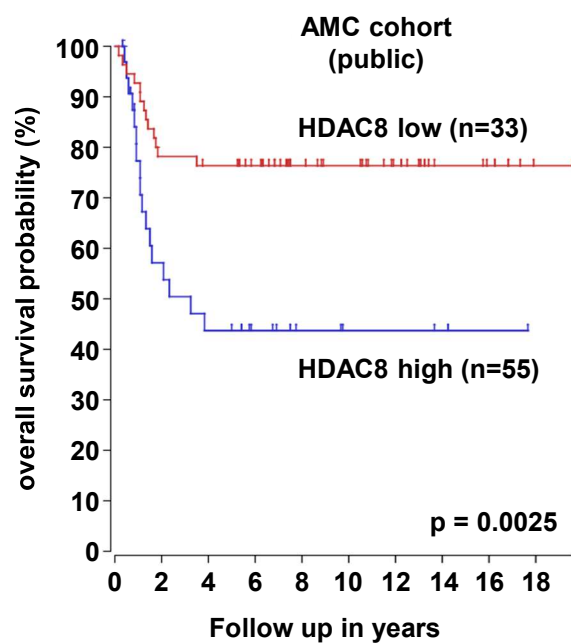**C**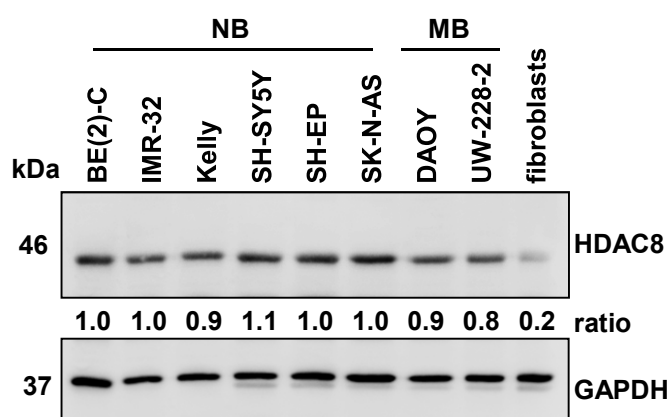**D**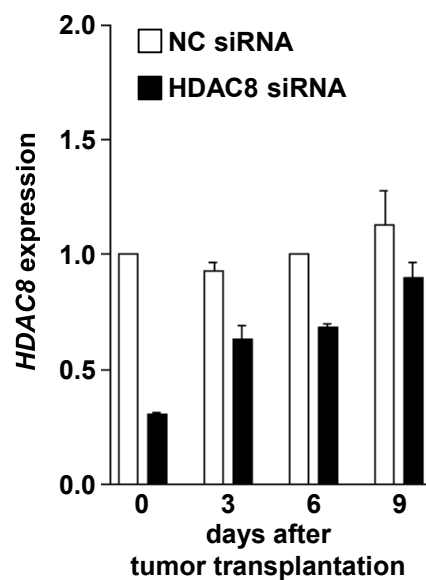

Supplement: Supplementary Figure S1 [file cddis201524x4.pdf]

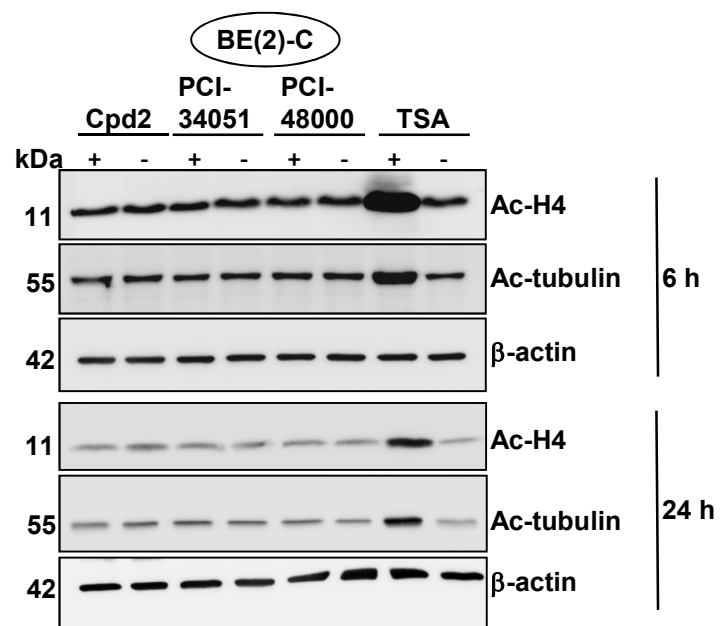

Supplement: Supplementary Figure S2 [file cddis201524x5.pdf]

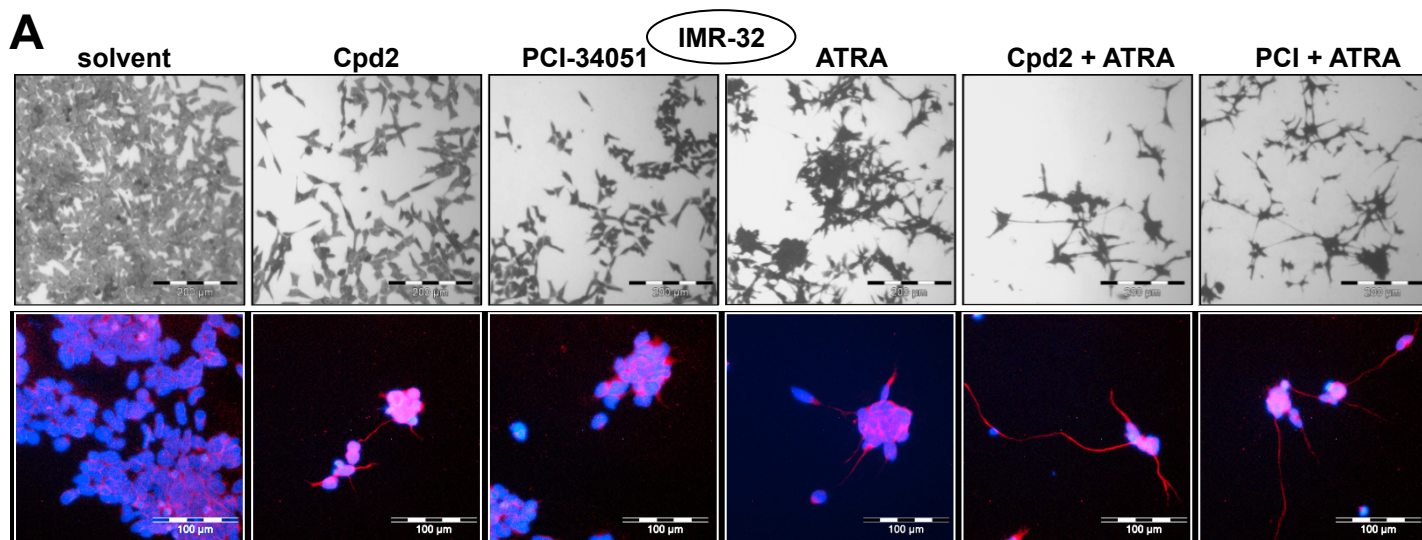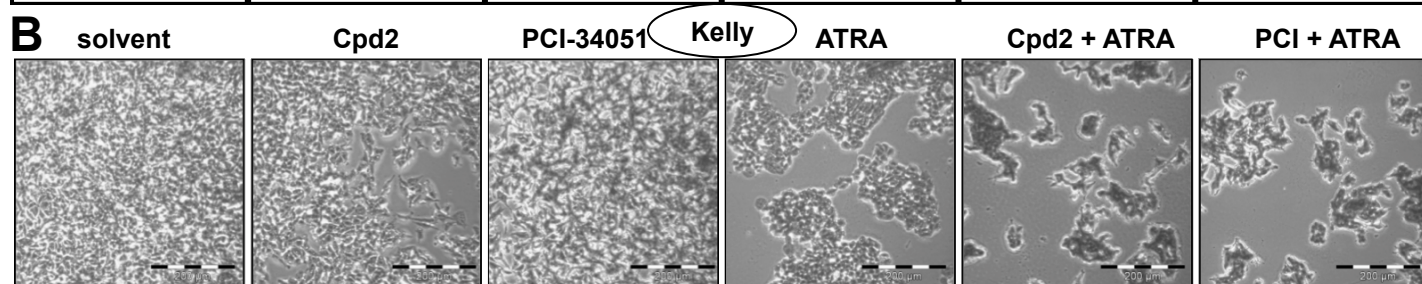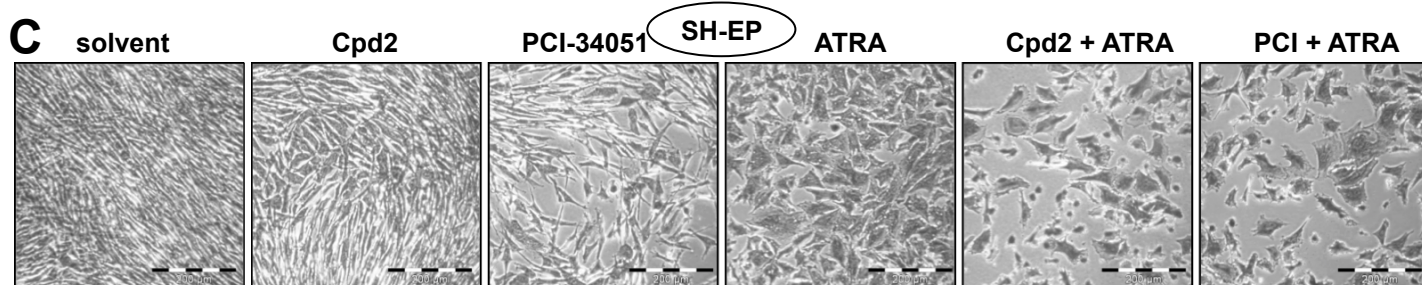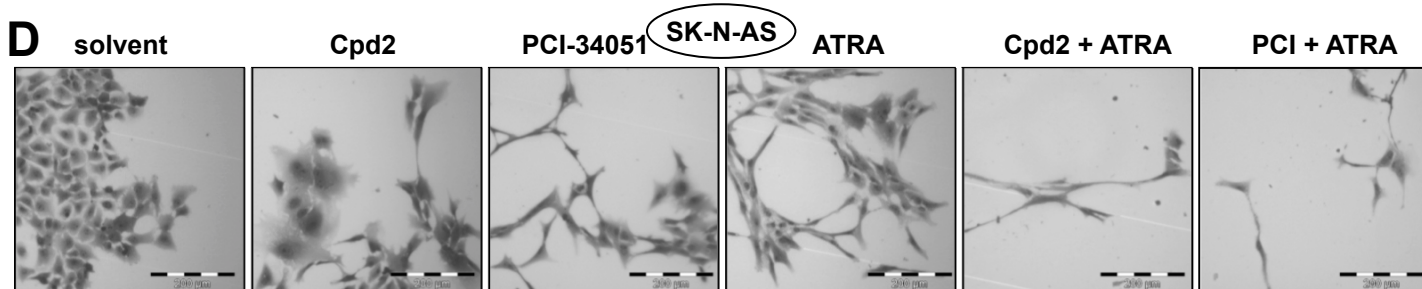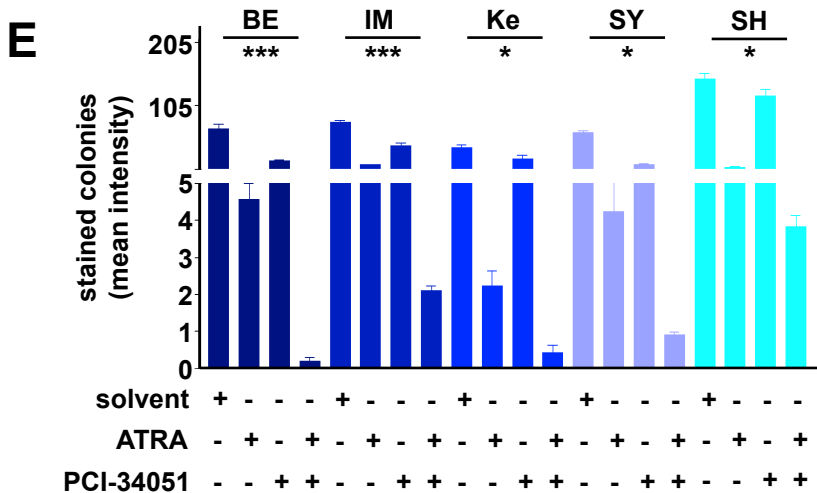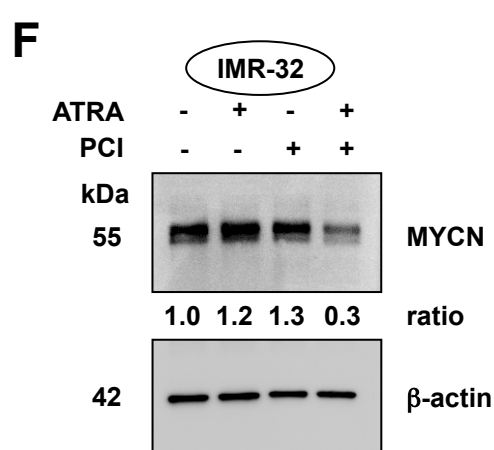

Supplement: Supplementary Figure S3 [file cddis201524x6.pdf]

**A**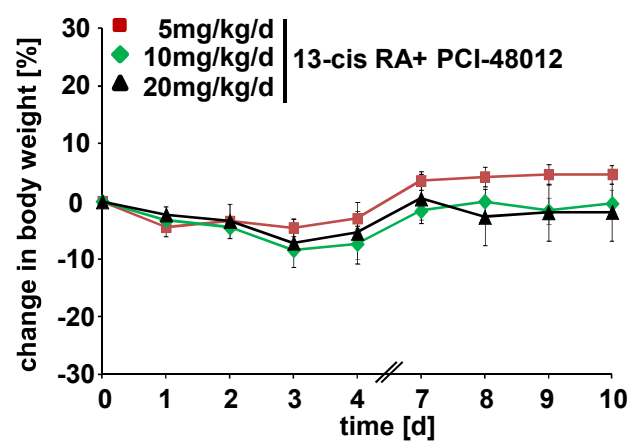**B**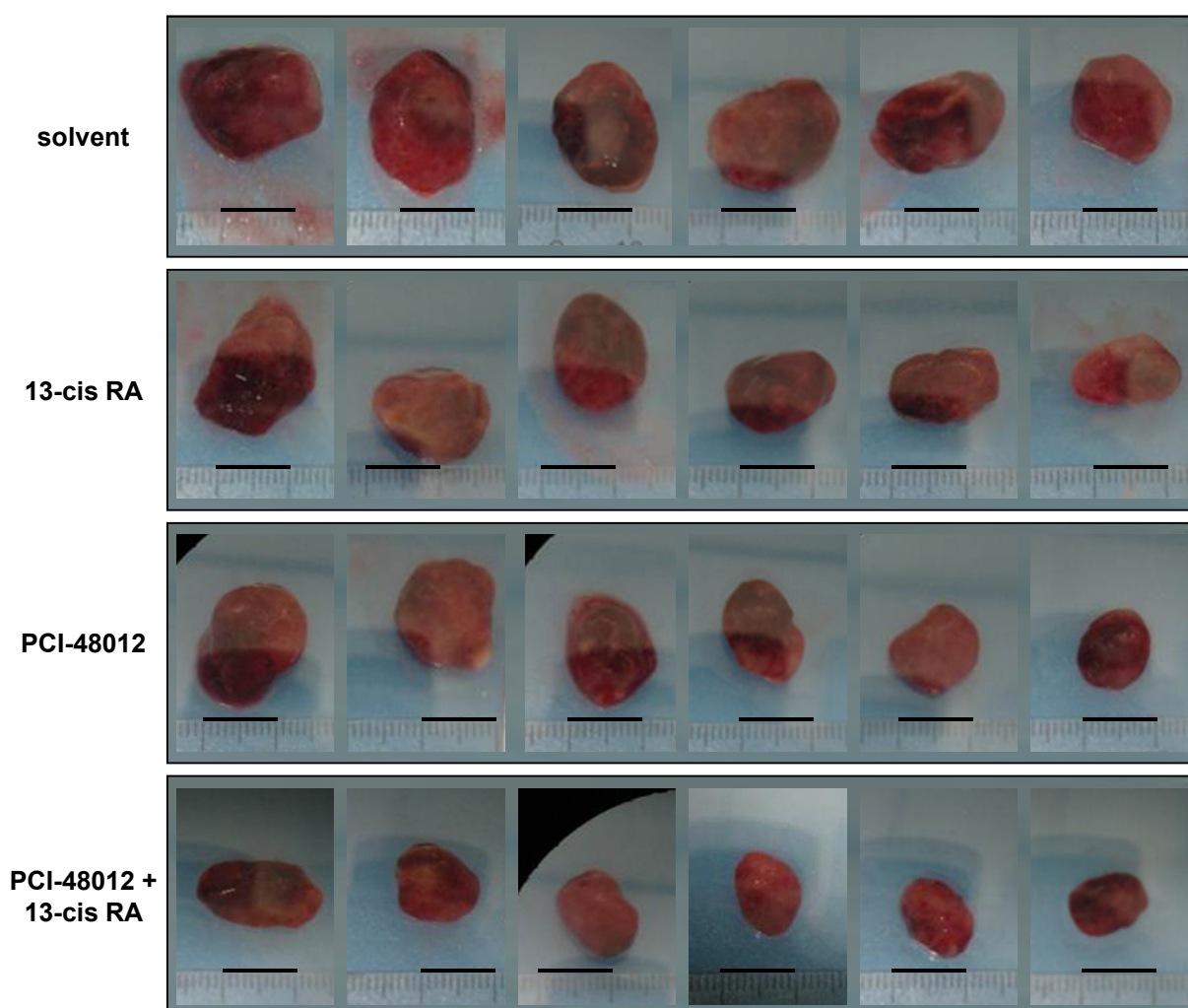

Supplement: Supplementary Figure S4 [file cddis201524x7.pdf]
